# A Review: Molecular Aberrations within Hippo Signaling in Bone and Soft-Tissue Sarcomas

**DOI:** 10.3389/fonc.2015.00190

**Published:** 2015-09-02

**Authors:** Michael D. Deel, Jenny J. Li, Lisa E. S. Crose, Corinne M. Linardic

**Affiliations:** ^1^Division of Hematology-Oncology, Department of Pediatrics, Duke University School of Medicine, Durham, NC, USA; ^2^Duke University School of Medicine, Durham, NC, USA; ^3^Department of Pharmacology and Cancer Biology, Duke University School of Medicine, Durham, NC, USA

**Keywords:** Hippo, sarcoma, osteosarcoma, Ewing sarcoma, rhabdomyosarcoma, mesenchymal, targeted therapy, pediatric cancers

## Abstract

The Hippo signaling pathway is an evolutionarily conserved developmental network vital for the regulation of organ size, tissue homeostasis, repair and regeneration, and cell fate. The Hippo pathway has also been shown to have tumor suppressor properties. Hippo transduction involves a series of kinases and scaffolding proteins that are intricately connected to proteins in developmental cascades and in the tissue microenvironment. This network governs the downstream Hippo transcriptional co-activators, YAP and TAZ, which bind to and activate the output of TEADs, as well as other transcription factors responsible for cellular proliferation, self-renewal, differentiation, and survival. Surprisingly, there are few oncogenic mutations within the core components of the Hippo pathway. Instead, dysregulated Hippo signaling is a versatile accomplice to commonly mutated cancer pathways. For example, YAP and TAZ can be activated by oncogenic signaling from other pathways, or serve as co-activators for classical oncogenes. Emerging evidence suggests that Hippo signaling couples cell density and cytoskeletal structural changes to morphogenic signals and conveys a mesenchymal phenotype. While much of Hippo biology has been described in epithelial cell systems, it is clear that dysregulated Hippo signaling also contributes to malignancies of mesenchymal origin. This review will summarize the known molecular alterations within the Hippo pathway in sarcomas and highlight how several pharmacologic compounds have shown activity in modulating Hippo components, providing proof-of-principle that Hippo signaling may be harnessed for therapeutic application in sarcomas.

## Introduction

### Overview of pediatric sarcomas

Sarcomas account for ~1% of all malignancies, but occur with higher frequency in children compared to adults, comprising ~15% of all childhood malignancies (1). The mainstay of treatment includes combining primary tumor control with surgery and/or radiation and systemic chemotherapy. While survival rates for localized sarcomas have improved to >70%, children with metastatic or recurrent disease continue to have dismal outcomes (2, 3).

Malignant bone and soft-tissue sarcomas arise in connective tissues (including bone, fat, muscle, blood vessels, deep skin tissues, nerves, and cartilage) and represent a histologically and molecularly heterogeneous group of tumors. Although the precise cell of origin of most of these tumors is not known, sarcomas are thought to develop as a result of genetic alterations in mesenchymal progenitor cells. While older adult patients often develop sarcomas with complex genetic karyotypes, there are relatively few genetic mutations driving tumorigenesis for the majority of childhood sarcomas, with the exception of some characteristic chromosomal translocations. In cases where the underlying molecular pathogenesis has been identified, this has not translated into improvements in survival rates for those patients with advanced or aggressive tumors, as many of the molecular drivers have not been able to pharmacologically modulated (2, 3). Discovering therapeutically targetable proteins that may be collaborating with such tumorigenic drivers is a promising new frontier for molecular oncology.

### Overview of Hippo signaling

The delineation of the Hippo pathway began in 2003 with identification of the *Drosophila* *hippo* gene. Hippo loss-of-function phenotypes were described concurrently by the Pan and Hariharan laboratories while screening for genes that negatively regulate tissue growth (4, 5). Subsequent studies unveiled Hippo signaling as an evolutionarily conserved cascade consisting of adaptor proteins and inhibitory kinases that regulate Yorkie, a pro-growth transcriptional regulator (6–8). Hippo signaling is highly conserved between *Drosophila* and mammals, and homologous pathway components across species are well described (9, 10). For this review, focus will be on mammalian Hippo signaling.

**Figure 1:**
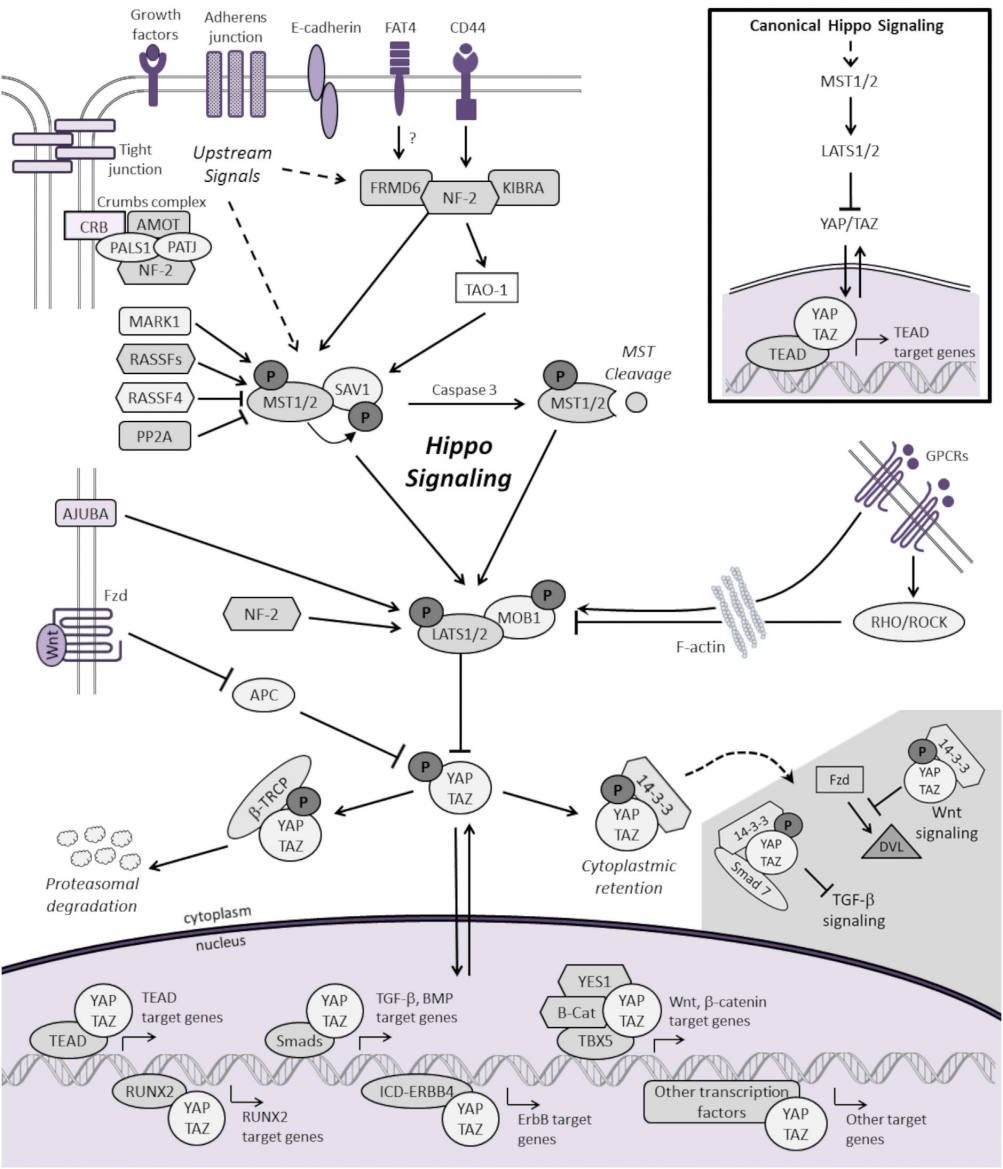
**Schematic representation of the mammalian Hippo signaling cascade**. Canonical Hippo transduction involves MST1/2 and LATS1/2 kinases, which, in conjunction with SAV1 and MOB1, phosphorylate, and inhibit the transcriptional co-activators YAP and TAZ. Regulation of YAP and TAZ are governed by plasma membrane proteins, cytoskeletal adaptor proteins, regulatory cross-talk from other signaling pathways, and intrinsic and extrinsic mechanical cues with the actin cytoskeleton. For simplicity, not all the known protein–protein interactions and regulators of Hippo signaling are represented. When Hippo signaling is “OFF”, YAP/TAZ translocate to the nucleus to serve as transcriptional co-activators for TEADs as well as other transcription factors (only a few of which are represented here) involved in cellular proliferation, differentiation, self-renewal, and apoptosis. See text for additional details.

As shown in Figure 1, the mammalian Hippo pathway relays plasma membrane and cytoplasmic signals into the nucleus, where it regulates the expression of a diverse group of target genes that control essential cellular processes, including proliferation, differentiation, and apoptosis. Canonical Hippo transduction involves serine/threonine kinases mammalian STE20-like protein kinase 1/2 (MST1/2, which are homologs of *Drosophila* Hippo) (4, 5, 11, 12) and large tumor suppressor homolog 1/2 (LATS1/2) (7, 13, 14), which, in conjunction with adaptor proteins Salvador homolog 1 (SAV1) (12) and Mob kinase activator 1 (MOB1) (15), phosphorylate and inhibit the transcriptional co-activators Yes-associated protein 1 (YAP, a homolog of Yorkie) and transcriptional co-activator with PDZ-binding motif (TAZ) [also known as WW domain-containing transcription regulator 1, WWTR1] (16). The Hippo pathway is “ON” when MST1/2 and LATS1/2 kinases are active. Through an interaction between the PPxY (PY) motifs of LATS1/2 and the WW domains of YAP and TAZ, activated LATS1/2 lead to phosphorylation of YAP and TAZ, which results in YAP/TAZ cytoplasmic retention and β-TRCP (β-transducin repeat-containing E3 ubiquitin protein ligase)-dependent proteasomal degradation (9, 10). When Hippo signaling is inactive or “OFF”, YAP and TAZ are localized to the nucleus, where they serve as transcriptional co-activators for TEA domain-containing sequence-specific transcription factors (TEADs) (17–21) as well as other transcription factors (16).

## Regulation of the Hippo Pathway

Much of our understanding of Hippo regulation comes from studies performed in epithelial tissue. In this context, the transcriptional activities of YAP and TAZ are regulated by four interconnected inputs: (1) plasma membrane proteins, which complex with YAP and TAZ directly to sequester them at cell–cell junctions; (2) upstream adaptor proteins, which activate core Hippo kinases to ultimately phosphorylate and repress YAP and TAZ; (3) regulatory cross-talk from other signaling pathways; and (4) intrinsic and extrinsic mechanical forces within the cell, which exert local control over YAP and TAZ localization. An overview of Hippo regulation is summarized below. For more detail, see the review by Grusche and colleagues (22), as well as three recent proteomic analyses that identified key protein–protein interactions with Hippo kinases, and YAP and TAZ within the global signaling network (23–25).

### Regulation through plasma membrane proteins

Growth control is signaled through plasma membrane proteins to upstream Hippo proteins, often in response to increased cell density. The Crumbs polarity complex, other polarity proteins, and adherens junctions, which all modulate each other, contribute inputs to various Hippo components (22, 26). E-cadherin and the junction-associated Ajuba protein family modulate MST and LATS kinases, respectively. The Crumbs complex involves transmembrane proteins that recruit scaffold proteins that localize to apical junctions and mediate cell polarity (27, 28). G-protein-coupled receptor (GPCR) ligands have been identified as regulators of Hippo signaling (29). Depending on the coupled G-protein, LATS1/2 kinases can either be activated or inhibited. YAP and TAZ directly influence the GPCR transcriptional activity, as YAP/TAZ are required for the expression of many GPCR-mediated target genes (29). The transmembrane hyaluronate receptor CD44 interacts with neurofibromin 2 (NF2, also known as Merlin) and other scaffold proteins to recruit LATS to the cell membrane, where it is phosphorylated (9, 30–32). Finally, the atypical cadherin protein Fat (*Drosophila*) is required for localization of Expanded (FRMD6 in mammals) to apical junctions, which results in activation of Hippo (MST1/2) (33). In avian cells, FAT4 has been shown to inhibit *YAP1*-mediated neuroprogenitor cell proliferation and differentiation (34).

### Regulation through upstream intracellular adaptor proteins

The core Hippo pathway is controlled by a complex upstream regulatory network. MST and LATS kinase activity are regulated by several upstream proteins, including Ras-association domain-containing family proteins (RASSFs1-10) (35, 36), kidney and brain protein (KIBRA) (37–39), thousand and one amino acid protein 1 (TAO-1) (40), MAP/microtubule affinity-regulating kinase 1 (MARK1) (41), and NF2. Via their interaction through a homologous SARAH (SAlvador–RAssf–Hpo) binding domain, RASSFs and SAV1 regulate MST activity (42). MST1/2 complexes with SAV1 to directly phosphorylate LATS1/2. MST1/2 bound to SAV1 can also bind to and phosphorylate MOB1, which binds LATS1/2 to promote autophosphorylation. While a growing inventory of functional interactions between upstream proteins and Hippo kinases are well described, the degree to which their binding is dependent on tissue type or cellular context, as well as their reliance on canonical Hippo signaling, requires further investigation. Several of the aforementioned proteins can also directly alter YAP activity in a manner independent of MST and/or LATS kinases (31, 43).

The Hippo pathway plays a major role in arbitrating cell contact inhibition, cell proliferation, and promoting apoptosis (44). As cells increase in confluence, the tumor suppressor NF2 localizes near cell junctions to activate Hippo signaling (45, 46). YAP suppression has been shown to rescue the hyperproliferative phenotypes caused by NF2 inactivation in both mesothelioma (47) and meningioma (48). Furthermore, overexpression of a dominant-negative TEAD suppressed the tumor growth resulting from liver-specific NF2 deletion in mice (49). A negative feedback loop between YAP/TAZ and LATS2 has also been described. YAP and TAZ stimulation and TEAD binding induces LATS2 expression, both directly and by inducing NF2 (50). In addition, YAP and TAZ may negatively regulate each other. For example, Taz accumulates in the livers of Yap knockout mice, while either *in vitro* suppression or overexpression of Yap results in inverse changes to Taz protein expression (50).

### Regulation through cross-talk with other pathways

Cell status and function, as well as overall tissue and organismal growth, is governed by an integrated network of morphogenic signals. Hippo transduction is proving to be a hub for such integration (51–53). Although studies are needed to clarify intra-pathway cross-talk in sarcomas, many of these pathways have been individually implicated in sarcomagenesis. YAP and TAZ are well recognized as co-activators for transcription factors of numerous signaling cascades. The specific ways in which signaling networks synergize or antagonize Hippo to coordinate biologic activity is only beginning to be understood. We highlight a few examples of regulatory cross-talk and refer to the studies referenced in Table 1 for additional details.

**Table 1:** **Pathway cross-talk with Hippo signaling**.

Pathway cross-talk	Reference
Developmental pathways	
Wnt/β-catenin	(67–70)
TGF-β	(60, 61, 71–74)
Notch	(67, 75–77)
Hedgehog	(78–80)
MAP kinase related	
MAPK/Erk	(81–83)
GPCRs	(29, 84, 85)
SAPK/JNK	(86, 87)
ErbB tyrosine kinases	(88)
PI3K/mTOR/Akt	(41, 89–91)
Jak/Stat	(92, 93)
Ras	(94–96)
Sox2	(97, 98)
MMP family	(99)
Mevalonate pathway	(100, 101)
Cellular metabolism	(102, 103)
Epigenetic modification	(104)
Cell cycle/CDK1	(105)

One example is illustrated by the relationship between the WNT and Hippo pathways. WNT activity is critical in myogenesis (54) and osteogenesis (55), and has recently been shown to be important in sarcomagenesis as well (56, 57). Rosenbluh et al. performed genome-scale loss-of-functions screens on 85 cancer cell lines (including osteosarcoma) and determined that WNT-active cancers are dependent upon β-catenin forming a complex with YAP and the transcription factor TBX5 to promote transcription of anti-apoptotic genes that are essential for cancer cell transformation and survival (58). This relationship was validated in a β-catenin-derived orthotopic colon cancer murine model, where Yap was required for tumor formation (58). In another study using murine cardiac muscle, knockdown of Hippo components Sav1, Mst1/2, or Lats2 results in increased Yap activity and cardiomyocyte proliferation with phenotypic cardiomegaly. Gene profiling from these mice reveal an elevated WNT signature, and the phenotypic effects could be offset by conditional loss of one β-catenin allele (59).

TGFβ and Hippo signaling also collaborate to direct cell behavior. YAP and TAZ associate with SMADs to promote transcription of TGFβ and BMP target genes (60–62). TGFβ signaling alters YAP/TAZ expression to drive mesenchymal stem cell (MSC) fate. For example, treatment of MSCs with BMP2 leads to increased TAZ expression and enhanced interaction with RUNX2 to promote osteoblast differentiation (63). Notch and Hippo signaling provide another example of coordinated cross-talk. Notch has been shown to be a driver of both bone and soft-tissue sarcomas (64–66). While no studies have examined the interplay of Notch and Hippo in sarcomas, overexpression of *Yap1* in mouse intestinal epithelia stimulates Notch signaling and the expansion of undifferentiated progenitor cells. However, treatment with γ-secretase inhibitors to block Notch signaling prevents the intestinal dysplasia caused by YAP (67). Together, these insights provide a deeper appreciation for the complex molecular circuitry that regulates Hippo activity in cell biology and malignancy.

### Cytoskeletal regulation through mechanical influences

To sustain proper function, from facilitating organ development during embryogenesis to maintain homeostasis postnatally, cells must perceive their microenvironment and respond appropriately to stimuli. In addition to transmitting biochemical signals, cells also extract information from mechanical cues. Mechanotransduction is the ability to perceive and translate physical stimuli [elasticity of the extracellular matrix (ECM) and forces exerted by cell–cell or cell–matrix interactions] into biochemical signals on a cellular level. Cells adapt to changes in tension through rapid cytoskeletal remodeling (106–108). YAP and TAZ have emerged as dynamic factors linking remodeling to nuclear transcriptional outputs that control cell behavior. Thus, by modulating YAP/TAZ activity, mechanical stimuli can direct cell fate and guide stem cell maintenance, proliferation, and differentiation (107, 109–111). For example, in *Drosophila*, the tension modulated within the cytoskeleton causes proportionate changes in wing growth through an Ajuba-Warts (homolog of LATS) complex (112).

**Figure 2:**
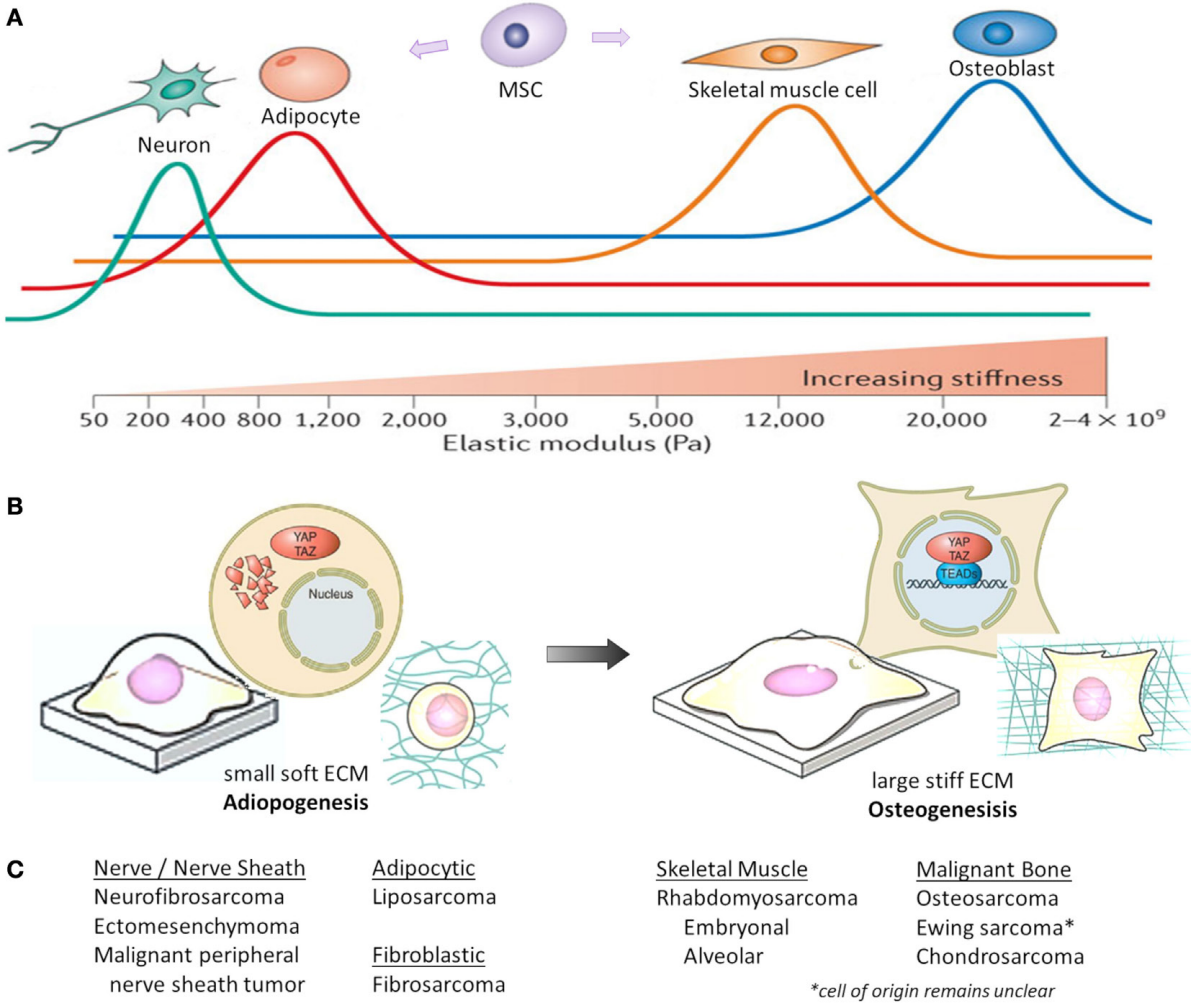
**Mechanical and physical influences on MSC cell fate**. Cell geometry and ECM stiffness regulate MSC lineage commitment into neurons, adipocytes, skeletal muscle cells, or osteoblasts. **(A)** Increasing ECM stiffness *in vitro* (by increasing type I collagen concentration and crosslinking) compromises tissue organization, inhibits apoptosis and lumen formation, and destabilizes adherens junctions. Through modeling different ECM elasticities *in vitro*, MSCs differentiate into the varying lineages at elasticities that recapitulate the physiological ECM stiffness of their corresponding natural niche (shown as colored lines, with peaks indicating maximal differentiation). Pa, Pascal. **(B)** When MSCs are either cultured on a soft matrix or are manipulated into a small round shape, YAP/TAZ remain inhibited in the cytoplasm and MSCs differentiate into adipocytes. However, when MSCs are either grown on a stiff matrix or stretched and manipulated into a “spread-out” morphology, YAP/TAZ localize to the nucleus as MSCs differentiate into osteoblasts. **(C)** Corresponding histologic sarcoma subtype [2013 WHO classification (230)], which may reflect varying lineage differentiation from mesenchymal progenitor cells. This represents only a theoretical link between mechanotransduction influencing mesenchymal progenitors and sarcoma, and not all sarcoma subtypes are represented. Figures **(A,B)** are modified with permission from Halder et al. (108) and Piccolo et al. (117).

In situations of high mechanical stress and low cell confluence, YAP and TAZ are transcriptionally active, resulting in proliferation and tissue growth. However, with increasing cell contact, adhesion molecules stimulate LATS activity, resulting in YAP/TAZ phosphorylation and nuclear exclusion (44). Both F-actin polymerization and stress fiber formation lead to the nuclear localization and activation of YAP/TAZ, whereas disrupting F-actin inhibits YAP/TAZ transcriptional activity (113–116). As shown in Figure 2, ECM stiffness and cell shape/spreading can also regulate YAP/TAZ localization by regulating the activity of Rho-GTPases and the formation of stress fibers and actin bundles (106, 110, 113). In MSCs, YAP and TAZ act as both sensors of mechanotransduction and mediators of cellular responses to mechanical signals (117, 118). YAP and TAZ remain inactive in the cytoplasm and direct MSCs to differentiate into adipocytes when human MSCs are exposed to low ECM stiffness, are cultured on a soft matrix, or are manipulated into a small round shape. However, YAP and TAZ are active in the nucleus and MSCs differentiate into osteoblasts when they are subjected to high ECM stiffness, are grown on a stiff matrix, or are stretched and manipulated into a “spread-out” morphology (119, 120). This mechanical control over YAP/TAZ activity supersedes density cues from cell–cell or cell–matrix contact (113, 115).

Interestingly, manipulation of YAP/TAZ expression can overrule mechanical influences to direct differentiation. When YAP/TAZ is suppressed, MSCs grown on a stiff ECM will undergo adipogenic differentiation. However, when activated YAP is overexpressed, MSCs grown on a soft ECM will undergo osteogenic differentiation (113). Knockdown of LATS1/2 has almost no effect on YAP/TAZ regulation by mechanical cues, and LATS-insensitive TAZ still responds to mechanical cues (113). Therefore, cellular mechanical stress can directly impact proliferation and tissue growth through YAP/TAZ, independent from Hippo signaling. Together, these studies emphasize the importance of cytoskeletal regulation of YAP and TAZ transcriptional activity, and demonstrate that YAP and TAZ are required for mechanical signals to direct MSC fate.

### Summary of hippo regulation

In summary, while the mechanistic and functional interactions between Hippo signaling and other regulatory pathways and cellular processes are not entirely understood, it is apparent that Hippo transduction links cell density and cell contact cues to morphogenic signals that regulate cell behavior. During development and tissue regeneration, the tumor suppressor function of Hippo signaling serves to offset the proliferative effects of other pathways. However, during malignant transformation, Hippo transduction is suppressed as cells evade contact inhibition, allowing the downstream effectors, YAP and TAZ, to co-activate TEADs as well as other transcription factors, to promote pro-proliferative and anti-apoptotic properties.

## Hippo Signaling in Mesenchymal Stem Cell Fate

While the precise cellular origin for most sarcomas remains uncertain, they are presumed to arise from mesenchymal precursors that fail to undergo terminal differentiation. These precursors have stem-like characteristics, including high proliferative and self-renewal potential. Therefore, insight into MSC regulation, lineage commitment, and differentiation (121), may shed light on sarcoma biology. As shown in Figure 2C, sarcoma subtypes are histologically described by the features of their presumed mesenchymal lineage. Summarized below are the known roles of Hippo signaling in modulating normal bone (osteogenic), fat (adipogenic), and muscle (myogenic) development, which are the origins of the most common sarcomas. YAP/TAZ are also critical mediators of cancer stem cell biology, a topic reviewed by others (122).

### Hippo signaling in osteogenic differentiation

Osteogenic differentiation is coordinated by the transcription factor, RUNX2, and a host of co-regulators (123), which activate the expression of osteoblast-specific genes, including osteocalcin (63, 124, 125). Through direct binding of the TAZ WW domain to the PY motif on RUNX2, TAZ has been identified as a transcriptional co-activator of RUNX2. Expression of an active TAZ mutant enhances RUNX2-driven gene expression two to threefold (63, 126), while knockdown of TAZ in MSCs inhibits osteogenesis when the cells are cultured under conditions favoring osteoblast differentiation (63). Transgenic mice with osteoblast-specific overexpression of Taz have significantly higher whole body bone mineral density, increased bone formation, and higher expression of RUNX2, osteocalcin, ALP, and osterix (127). TAZ-mediated osteogenesis may also occur downstream of the WNT pathway, since WNT3A can cause PP1A-mediated TAZ dephosphorylation, leading to TAZ nuclear localization and induction of osteogenic differentiation (68).

While the role of TAZ in supporting osteogenesis is clear, the role of YAP is more complex. When an activated YAP mutant was overexpressed in MSCs, osteogenic differentiation was promoted over adipogenic differentiation, even under conditions favoring the latter (113). However, YAP can also act as repressor of RUNX2 when it is regulated by non-canonical pathways (128). For example, when Src/Yes tyrosine signaling is inhibited, Yap tyrosine phosphorylation is blocked, Yap dissociates from RUNX2, and osteocalcin is induced (128, 129). Last, there is evidence that YAP is a direct target of SOX2, a transcription factor important for MSC cell fate; in situations of high SOX2 or YAP expression, osteogenesis is blocked, while depletion of either SOX2 or YAP enhances osteogenesis (98).

In addition to YAP/TAZ, there is evidence that upstream scaffold proteins influence osteogenesis. *Rassf2* knockout mice develop bone-remodeling defects, and *in vitro* studies show that ablation of RASSF2 suppresses osteoblastogenesis while promoting osteoclastogenesis (130).

### Hippo signaling in adipogenic differentiation

A key transcription factor orchestrating adipogenesis is peroxisome proliferator-activated receptor gamma (PPARγ), which contains a PY motif for binding the WW domains on YAP and TAZ (63). In this context, binding of TAZ has an inhibitory role, suppressing transcriptional activity. When cultured under conditions that promote adipogenic differentiation, knockdown of TAZ permits MSCs to differentiate toward this lineage (63). Similarly, treatment with the small molecule KR62980 (a ligand for PPARγ that antagonizes adipocyte differentiation) does so by promoting TAZ nuclear localization and enhanced interaction between TAZ and PPARγ (131).

Recent work has shed light on the role of YAP in adipogenesis. Similar to osteogenesis, YAP is downstream of SOX2. However, YAP levels must be fine-tuned; both over or under-expression of YAP inhibits adipogenesis. Mechanistically, YAP induces the Wnt antagonist Dkk1 to diminish osteogenic signaling in favor of adipogenesis. In addition to YAP and TAZ, upstream Hippo regulators have been implicated. The Hippo adaptor protein SAV1 contains WW domains that can interact with the PY motif within PPARγ (132). MST1/2 stimulated SAV1 to bind PPARγ, which stabilizes and increases PPARγ levels, ultimately leading to adipogenic differentiation. In addition, knockdown of MST1/2 or SAV1 results in the inhibition of adipogenesis (132), though it is not known whether this effect is through canonical Hippo transduction or an alternate pathway.

### Hippo signaling in myogenic differentiation

Myogenic differentiation is driven by the myogenic regulatory factor family [MRFs: MyoD, myogenin, MRF4, and myogenic factor 5 (Myf5)]) (133–135) in coordination with myocyte-specific MEF2 enhancer factors (136, 137). In murine C2C12 skeletal muscle myoblasts, YAP supports an undifferentiated phenotype and promotes myoblast proliferation (138–140). Upon differentiation, nuclear YAP is translocated to the cytoplasm, with a 20-fold increase in YAP phosphorylation. Overexpression of YAP S127A, a mutant that cannot be phosphorylated at the LATS-regulated site, impedes myotube formation, and alters the expression of MRFs (139). Activation of YAP causes upregulation of Myf5, which promotes myoblast proliferation. Activated YAP also leads to down-regulation of MyoD and MEF2, which are important in cell-cycle exit and differentiation, as well as upregulation of inhibitors of MyoD and MEF2, such as ID2, Twist1, and Snai1/2 (133, 138). In activated satellite cells, which are resident stem cells of skeletal muscle, high YAP activity prevents differentiation and promotes proliferation (138, 140). YAP suppression dramatically reduces satellite cell-derived myoblast proliferation (140). Additionally, muscle CAT (MCAT) elements, which are TEAD-binding sites, are found in the promoters of genes that are selectively expressed in terminally differentiated skeletal muscle (140, 141).

Interestingly, while YAP inhibits myogenic differentiation, some studies suggest TAZ may enhance myogenesis. TAZ physically binds MyoD to enhance binding to the myogenin gene promoter to activate MyoD-dependent gene transcription (142, 143). Ectopic overexpression of TAZ in C2C12 myoblasts results in accelerated myofiber formation, whereas TAZ loss lessened myogenic differentiation (142).

Evidence of upstream Hippo pathway regulators in muscle differentiation is limited. However, MST was found to have a pro-differentiation role during an investigation of caspase 3 in myogenesis (144). While caspases are classically known for their role in apoptosis, non-apoptotic functions have been reported. This appears to be the case in myogenesis, as caspase 3 was robustly activated in differentiating myoblasts without inducing apoptosis. Caspase 3-deficient myoblasts or C2C12 cells treated with caspase inhibitors are less able to differentiate, in part due to caspase 3-mediated regulation of MST1. Additionally, MST1 is a substrate for caspase 3, and cleaved MST1 was enriched in myoblasts undergoing differentiation. In caspase 3-deficient myoblasts, introduction of the cleaved MST1 induced myogenic differentiation, proving a link between these two pathways. However, MST1 activation must be tightly controlled, as MST1 activation in wild-type myoblasts ultimately led to cell death (144). While this study suggests a role for MST1 in myogenic differentiation, connections between MST1 activation by caspase 3 and the canonical Hippo pathway in muscle remain to be determined.

## The Molecular Basis for Hippo Signaling in Sarcomas

Sarcomas comprise a group of clinically and histologically diverse tumors of mesenchymal origin. They can develop anywhere in the body, with about half arising in bone and half in soft tissues. In children and adolescents, osteosarcoma (OS) and Ewing sarcoma (EWS) are the two most common malignant bone sarcomas, while rhabdomyosarcoma (RMS) and non-rhabdomyosarcoma soft-tissue sarcomas (NRSTSs) are the major classes of malignant soft-tissue sarcomas (145).

As reviewed earlier, Hippo signaling is essential for proper organ growth, amplification of tissue-specific progenitor cells during tissue regeneration, and cellular proliferation (10, 146). In 2007, Dong and colleagues generated a liver-specific conditional *Yap1* transgenic mouse model that develops hepatocellular carcinoma (10). This led to the understanding that YAP is important in cancer and identified Hippo signaling as a tumor suppressor pathway in mammals. In other genetically engineered mouse models (GEMMs), mutations or altered expression of Hippo pathway genes gives rise to sarcomas, substantiating Hippo pathway deregulation in sarcomagenesis (138, 147–149). The next section will review the molecular basis of dysregulated Hippo signaling in bone and soft-tissue sarcomas. Each subsection will highlight the pro-tumorigenic role of YAP/TAZ, with subsequent cataloging of other Hippo pathway member involvement. Table 2 summarizes these alterations.

**Table 2:** **Proposed involvement of Hippo pathway components in sarcoma biology**.

Sarcoma type	Component	Summary of proposed pathologic role	Reference
Osteosarcoma	YAP	*YAP1* expression is elevated and correlates with tumor staging and an increase in Hippo target genes Suppression of YAP promotes differentiation, and decreases cell proliferation and tumor growth YAP is a direct target of SOX2 in osteoprogenitors and *YAP1* expression is altered by SOX2 abundance OS transgenic mice with upregulated Hedgehog signaling display high *YAP1* expression The long non-coding RNA H19 is aberrantly induced by *YAP1* overexpression	(80, 153, 155)
	RASSFs	*RASSF5* is downregulated in human OS tumors and expression negatively correlates with metastasis *In vitro* overexpression of RASSF5 leads to decreased cell proliferation and invasion *RASSF10* promoter is epigenetically silenced through hypermethylation	(156–158)
	NF2	*NF2* expression is decreased and NF2 is shown to be a direct target of SOX2 in osteoprogenitors 63% of *Nf2*^+/−^ mice develop OS. Increased penetrance and decreased latency and survival with *Nf2^+/^*^−^*p53*^+/−^ mice. Both groups show loss of wild-type *Nf2* allele	(97, 147, 160, 198, 199)
	MOB1	24% of *Mob1A*^Δ^*^/^*^+^*1B^tr/tr^* or *Mob1A*^Δ^*^/^*^Δ^*1B^tr/^*^+^ mice develop extraskeletal OS in 25–70 weeks	(148)

Ewing sarcoma	YAP	*In vitro* YAP suppression decreases proliferation in EWS cells BMI-1 stabilization of YAP is proposed to be a means for EWS cells to overcome contact-inhibition	(163)
	RASSFs	Hypermethylation of *RASSF1A* and *RASSF2* occurs at high frequency and correlates with worse outcomes	(165, 166)

ERMS	YAP	*YAP1* is elevated in human tumors and correlates with increased proliferation and clinical outcomes Copy number gains of the *YAP1* locus are reported YAP suppression results in decreased proliferation and increased differentiation *Myf5-* or *Myod1-hYap1 S127A* mice generate ERMS tumors within 4–8 weeks after *Yap1 S127A* expression 100% of *Pax7-hYap1 S127A* mice generate ERMS-like tumors within 10–11 weeks after injury	(138, 178)

ARMS	YAP	*YAP1* expression is increased in human tumor samples *In vitro* suppression of YAP results in decreased proliferation and increased senescence	(138, 178)
	RASSF4	*RASSF4* is a PAX3-FOXO1 target gene Overexpression of RASSF4 promotes cell proliferation *In vitro* loss of RASSF4 leads to decreased cell growth	(178)

NRSTS	YAP	STSs display gene amplification and overexpression of *YAP1* with increased TEAD-associated genes YAP complexes with TEAD and the cell cycle transcription factor FOXM1 to support STS tumorigenesis	(186, 191)
	RASSF1A	*RASSF1A* hypermethylation is reported in ~20% of adult STSs and correlates with clinical outcomes	(189)
	MST1/2	Hypermethylation of *MST1* and *MST2* occurs in 37 and 20% of STS, respectively	(187, 200)
	LATS1/2	Hypermethylation of *LATS1* is associated with poorer prognosis and shorter survival times in human STS	(149, 188, 189)
		60% of *Lats1^−/−^* mice die *in utero* but 14.3% of surviving female *Lats1^−/−^* mice develop fibrosarcomas by 4–10 months. Adding carcinogen exposure decreases latency and increases penetrance to 83%	

Fibrosarcoma	MOB1	22% of *Mob1A*^Δ^*^/^*^+^*1B^tr/tr^* or *Mob1A*^Δ^*^/^*^Δ^*1B^tr/^*^+^ mice develop fibrosarcoma in 25–70 weeks	(148)
	NF2	7% of *Nf2*^+/−^ mice develop fibrosarcoma. 32% of *Nf2*^+/−^*p53*^+/−^ mice develop fibrosarcoma	(147)

EHE	TAZ-CAMTA1 YAP-TFE3	TAZ-CAMTA1 and YAP1-TFE3 fusion proteins are pathognomonic findings in EHE tumor samples	(193–195)

### Hippo signaling in osteosarcoma

Osteosarcoma is the most common primary malignancy of bone, with a 5-year overall survival of 60–70% (150). Given its decreased radiosensitivity compared to other sarcomas, surgical resection with chemotherapy is the mainstay of treatment. OS tumors are characterized by complex genomic rearrangements as well as copy number variations (151, 152). Mutations or loss-of-function of tumor suppressors *RB1* and *TP53* are two of the most common genetic alterations and are reported in ~50 and ~30% of tumors, respectively (151). Aberrations in Hippo signaling are proving to be important in the biology of OS.

#### YAP

Human tissue microarray analyses have revealed high YAP1 protein expression in OS compared to surrounding non-cancerous tissue, and expression correlates with staging (153). These findings corroborate other studies which showed high YAP1 expression in 78% of human OS samples and an increase in Hippo pathway target genes (80, 97, 154). Nuclear localization of Yap was found in Kios-5 murine OS cell lines, and Yap (and Taz, to a lesser extent) protein expression was also increased. *In vitro* suppression of Yap was associated with decreased cell proliferation and invasion, as well as decreased expression of Runx2, CyclinD1, and MMP-9. Decreased tumor growth was observed with *in vivo* Yap suppression in murine xenografts (155), as well as transgenic mouse models (80).

The mechanism of YAP upregulation in OS is complex but appears to be due in part to the stem cell transcription factor SOX2. In murine OS cell lines, Sox2 was found to directly repress the Hippo pathway activators, Nf2 and Kibra, leading to increased YAP. When grown as osteospheres, where stem cells are enriched, YAP expression was higher (and Nf2 lower) compared to adherent cells. In cells depleted of Sox2, either Yap overexpression or Nf2 suppression restored osteosphere formation. Conversely, suppressing Yap or overexpressing Nf2 promoted osteogenic differentiation and prevented osteosphere formation. The differentiated phenotype of OS cells induced by Nf2 could be overcome by either overexpressing wild-type or constitutively active mutant Yap, but not mutant Yap with a deficient TEAD-binding site. This regulation of Yap by Sox2 occurs through canonical Hippo signaling, as suppression of either Mst1/2 or Lats1/2 abolished Nf2-induced osteogenic differentiation as well as changes in Yap expression and function (97).

YAP can also be upregulated by Hedgehog (Hh) pathway activation. Malignant OS occurs with high penetrance in *Ptch1*^c/+^;*p53*^+/−^;HOC-Cre mutant mice, in which Hh signaling is partially upregulated in a p53 heterozygous background. Resultant tumors have high Yap1 expression, which is significantly reduced with Hh inhibition, and suppression of Yap1 blocks tumor progression. This same study showed that the Hh-Yap axis may regulate the expression of H19, a maternally imprinted long non-coding RNA implicated in tumorigenesis (80).

#### RASSFs

Two RASSFs (RASSF5 and RASSF10) have been implicated as tumor suppressors in OS. Similar to other RASSF family members, *RASSF5* and *RASSF10* are seen downregulated in human tumors (including OS) by CpG island promoter hypermethylation (156). In a human tissue microarray representing 45 OS samples, RASSF5 was significantly downregulated and expression negatively correlated with distant metastasis (157). In human U2OS cells, *in vitro* suppression of RASSF5 conveyed resistance to TNF-α-induced apoptosis, which is thought to occur through interaction and inactivation of the pro-apoptotic function of MST1 (158). Conversely, overexpression of RASSF5 in human OS cell lines decreases cell proliferation, increases apoptosis, and inhibits invasion.

#### NF2

In humans, germline or somatic mutations in one allele of *NF2* result in the disease neurofibromatosis type 2, which is associated with schwannomas, meningiomas, and ependymomas. However, mice heterozygous for *Nf2* develop a variety of malignant tumors at high frequency, including OS (63%). Somatic mutations of the wild-type *Nf2* allele were found in almost all of these tumors, implying that loss of heterozygosity of *Nf2* may be required for sarcomagenesis (147).

#### CD44

CD44 is a cell-surface glycoprotein that transmits extracellular signals to the ERK, AKT, and Hippo pathways (82, 159). CD44 was found to be suppressed by NF2, leading to decreased migration and invasion in OS cell lines *in vitro*, although an enhanced OS malignant phenotype was observed with knockdown of *CD44* in mice xenografts (160). Others have shown that NF2 mediates contact growth inhibition through ECM signals by complexing with CD44 (32).

#### MOB1

*In vitro* overexpression of MOB1A impairs cellular proliferation, while suppression of MOB1A leads to aberrant mitosis (15). In double-mutant mice lacking both *Mob1A* and *Mob1B*, complete loss of both alleles (*Mob1A*^Δ/Δ^*1B^tr^*^/^*^tr^*, null mutation of *Mob1A*, gene trap of *Mob1B*) is embryonically lethal. However, double-mutant mice retaining one allele of either (*Mob1A*^Δ/+^*1B^tr^*^/^*^tr^* or *Mob1A*^Δ/Δ^*1B^tr^*^/+^) survive and spontaneously develop tumors with 100% penetrance within 70 weeks. Extraskeletal OS arose in 24% (9/37) of mice, while benign exostosis occurred in 92% (34/37). All the tumors examined from either single heterozygote (*Mob1A*^Δ/+^*1B^tr^*^/^*^tr^* or *Mob1A*^Δ/Δ^*1B^tr^*^/+^) group revealed loss of the wild-type *Mob1* allele, suggesting loss of heterozygosity may be necessary for tumor growth (148).

### Hippo signaling in Ewing sarcoma

Ewing sarcoma is the second most common malignant bone tumor in children and young adults. Although the 5-year overall survival is about 70%, 30–40% of patients either present with metastatic disease or develop recurrence, where outcomes are worse (161). EWS is characterized by a t(11;22) chromosomal translocation, which generates a fusion gene encoding the EWS-FLI1 chimeric protein that is thought to be the predominant driver of EWS tumorigenesis (162). The molecular basis for dysregulated Hippo signaling in EWS is beginning to be studied, as summarized below.

#### YAP

YAP suppression in human EWS cell lines decreases proliferation and anchorage-independent colony formation (163). A relationship between YAP and BMI-1, a Polycomb complex protein involved in chromatin remodeling (164), has been proposed. In studies examining the effect of cell density in cultured EWS cells, loss of BMI-1 had no effect in low-density, while it caused cell-cycle arrest and death under conditions of confluence. These findings may be due in part to the role of BMI-1 in stabilizing YAP expression and activity, and may serve as a means for BMI-1-driven EWS cells to overcome contact inhibition (163).

#### RASSFs

Hypermethylation of the promoter regions of *RASSF1A* and *RASSF2* has been described in EWS and is correlated with worse clinical outcome (165, 166). One study of 55 human EWS tumors reported methylation rates for *RASSF1A* and *RASSF2* of ~52 and ~42%, respectively (165). In *in vitro* studies, overexpression of either RASSF1A or RASSF2 in EWS cells reduced their ability to form colonies (165). In a separate study, methylation of *RASSF1A* was observed in 75% (3/4) of EWS cell lines and 68% (21/31) of human tumors (166), though these studies are contradicted by other reports that did not demonstrate increased *RASSF1A* hypermethylation (167, 168). The EWS-FLI1 fusion protein has recently been shown to provoke widespread epigenetic changes, including altered DNA methylation, although it is not known whether there is a direct effect on *RASSF* expression (168, 169).

### Hippo signaling in rhabdomyosarcoma

Rhabdomyosarcomas are soft-tissue sarcomas and account for approximately 8% of all pediatric solid tumors (170). The two major histological subtypes are termed embryonal (ERMS) and alveolar (ARMS) rhabdomyosarcoma. ERMS, which is more common, typically arises in the head and neck or retroperitoneum of younger children and conveys a better prognosis (localized tumors have >70% 5-year overall survival) (171, 172). ERMS tumors demonstrate numerous chromosomal aberrations, including genomic amplifications, loss of heterozygosity of specific chromosomal regions, frequent chromosomal gains in 2, 8, 12, and 13, and loss-of-imprinting (171–174). ARMS make up about 25–30% of cases and usually arise in the extremities or trunk and occur more frequently in adolescents. ARMS is characterized by recurrent chromosomal translocations, principally t(2;13) and t(1;13), which result in the expression of PAX3-FOXO1 and PAX7-FOXO1 fusion proteins, respectively (175). These aberrant chimeric proteins are oncogenic transcription factors that confer a poor prognosis (5-year overall survival <15% for metastatic or recurrent tumors) (173, 175–177). Interestingly, fusion-negative histologic ARMS have a cytogenetic and molecular profile similar to ERMS, and correspondingly similar clinical behavior (177).

#### YAP

YAP protein is upregulated in both ERMS and ARMS tumors (138, 178). In ERMS and fusion-negative ARMS, this is due in part to increased *YAP1* locus copy number. The importance of YAP in ERMS was confirmed by the remarkable finding that expression of YAP S127A is sufficient for ERMS tumorigenesis in a GEMM (138). This finding was particularly surprising given prior work showing YAP1 S127A expression in adult mouse muscle caused atrophy (179). Similar to this study, limb stiffness and gait defects were the initial phenotypes observed in Myf5/MyoD-YAP1 S127A mice (138). However, analysis of their muscle beds found that within the muscle damage were sites of active muscle regeneration and expansion of mononucleated cells. These were confirmed to be ERMS lesions, as they stained positive for ERMS histological markers. Tumor cells from these mice were transplantable, leading to secondary ERMS tumors with short latency. Given the high proportion of mononucleated cells in the primary tumor, Tremblay and colleagues hypothesized that satellite cells could serve as an ERMS cell of origin in this model. While expression of YAP1 S127A in the Pax7 (satellite) cell lineage did not induce ERMS formation, YAP1 S127A did transform satellite cells in the context of muscle injury. This suggests that hyperactive YAP signaling in activated satellite cells has transformative properties.

Using this GEMM model, hyperactive YAP signaling in ERMS tumors was found to induce a myogenic differentiation block. When YAP S127A expression was reduced, tumors rapidly regressed, and tumor cells spontaneously expressed markers of terminal muscle differentiation. Similarly, endogenous YAP suppression in ERMS RD cell xenografts caused myogenic differentiation (138). These findings are in line with earlier work implicating a role for YAP signaling in regulating myogenic differentiation. In proliferating C2C12 and satellite cells, YAP levels are high and localized in the nucleus. Upon differentiation stimulus, YAP mRNA expression is reduced and YAP becomes cytoplasmic (139, 140). This suggests an important role for YAP signaling in maintaining a high proliferative and anti-differentiation state. Similarly, YAP S127A can block C2C12 and satellite cell *in vitro* differentiation. This differentiation block is believed to be due to transcriptional changes induced by YAP-TEAD, particularly through upregulation of pro-proliferative genes and repression of MYOD1 and MEF2 regulation of terminal differentiation genes (138).

Additional studies have supported a role for YAP in RMS. A subset of ERMS tumors harbor mutations in the *PKN1* gene (encoding a kinase of the protein kinase C superfamily), which imparts a gene expression signature associated with activated YAP (180). In ARMS cells, *in vitro* genetic suppression of YAP induces growth arrest and senescence (178).

#### RASSF4

A role for the Hippo pathway in ARMS began with the identification of *RASSF4* as a PAX3-FOXO1 target gene (178). Using transcriptional profiling studies, PAX3-FOXO1-expressing myoblasts were found to upregulate *RASSF4* expression. Further, PAX3-FOXO1-positive ARMS cell lines and human tumors had elevated *RASSF4* levels, and high *RASSF4* expression was associated with worse RMS clinical prognosis. Loss-of-function studies demonstrated that RASSF4 was promoting cell proliferation and senescence evasion in ARMS cells. These RASSF4 functions were due to inhibition of MST1 signaling to MOB1. While no changes in signaling to LATS1 were observed, RASSF4-deficient ARMS cells did express lower levels of YAP1 protein. However, cells expressing a hyperactive YAP1 (YAP S127A) could not reverse the phenotypes associated with RASSF4 loss, suggesting an indirect connection between RASSF4 and YAP signaling (178). Altogether, these studies suggest that suppression of MST1-MOB1 signaling is an important oncogenic function of RASSF4 in ARMS.

#### TEAD-NCOA2 Fusions

NCOA2 is a transcriptional co-activator for steroid and nuclear hormone receptors. Fusion of *TEAD* to *NCOA2* was found in tumor tissue removed from a 4-week-old child with spindle cell RMS (181), a rare variant of ERMS (182). While *NCOA2* gene rearrangements with other gene partners are seen in high frequency in congenital spindle cell RMS and mesenchymal chondrosarcomas (181, 183), the clinical and molecular significance of TEAD as a binding partner in this case is not known.

### Hippo signaling in non-rhabdomyosarcoma soft-tissue sarcomas

Non-rhabdomyosarcoma soft-tissue sarcomas comprise the fifth most common group of solid tumors in children, accounting for 8–9% of childhood malignancies. These are histologically heterogeneous tumors that share some biologic characteristics. Surgical resection results in remission for about 80% of patients presenting with localized disease, though survival for those with unresected or metastatic disease remains poor (184). Many NRSTS, particularly those common in children, are characterized by disease-defining chromosomal translocations. Examples include synovial sarcoma t(X;18) and alveolar soft part sarcoma t(X;17), which result in the SYT-SSX and ASPL-TFE3 oncogenic fusion proteins, respectively (145). Other NRSTSs that are more common in adults, such as leiomyosarcoma or undifferentiated sarcoma, display multiple complex karyotypic abnormalities with frequent mutations in the TP53 and RB tumor suppressor pathways (185).

#### YAP

Nuclear staining for YAP is increased in a subset of human STS samples, compared to corresponding normal connective tissue (186). KRAS-based [*LSLKras^G12D^*^/+^;*Tp53^fl^*^/^*^fl^* (KP)] GEMMs were used to further investigate the role of YAP in STS. *Yap* suppression in allograft tumors generated from KP cells results in decreased cell proliferation and tumor growth, and treatment with verteporfin to block the YAP–TEAD interaction decreased transcription of *Yap1* target genes. Many of the downregulated mRNAs in this model were noted to also be targets of *Foxm1*, a transcription factor involved in cell-cycle progression. FOXM1 is ordinarily inhibited by direct interaction with members of the TP53 and RB tumor suppressor pathways, and it is often overexpressed in malignancies where these tumor suppressor functions have been lost (186). FOXM1 expression was found to be increased in a variety of human sarcoma samples. In xenograft studies, FOXM1 suppression inhibited sarcoma growth. Co-immunoprecipitation and ChIP-seq experiments reveal that FOXM1 physically associates with a YAP/TEAD complex (186). YAP suppression in human sarcoma cell lines resulted in decreased proliferation and decreased FOXM1 expression, suggesting a novel role for YAP in co-activating FOXM1-mediated transcription in STS.

#### MST1/2

Hypermethylation of *MST1* and *MST2* promoters occurs in 37 and 20% of all STS (including RMS), respectively (187). In leiomyosarcoma samples, hypermethylation of *RASSF1A* and *MST2* were mutually exclusive, implying a common signaling pathway may exist for both genes. Surprisingly, methylation of the *MST1* promoter appears to correlate with a decreased risk of tumor-related mortality (187), albeit from a retrospective cohort with a small sample size.

#### LATS

Reduced *LATS* gene expression was observed in 14% (7/50) of human adult STS tumors (188). These findings correlate with subtype, as three of four myxoid liposarcomas, three of seven leiomyosarcomas, and one of nine malignant fibrous histiocytomas showed reduced or no expression of LATS1. In one of those samples, an allelic loss of the *LATS1* locus in chromosome 6q23-25.1, resulting from a missense point mutation, was observed. The other six samples showed aberrant hypermethylation of the putative *LATS1* promoter (188), corroborating another study showing hypermethylation of the *LATS1* promoter in 7% (3/43) of human STS samples (187). Hypermethylation of *LATS1* in STSs is associated with a worse prognosis and shorter survival times (189). It is not known whether epigenetic regulation of Hippo pathway kinases alters the expression of YAP and TAZ.

In transgenic mouse models, most mice (60/101) homozygous for a null mutation in *Lats1* died *in utero* or within post-natal day 1. However, ~14% of surviving female *Lats1*^−/−^ mice developed large NRSTS by 4–10 months of age consistent with fibrosarcomas. After exposure to the carcinogen DMBA and repeated exposure to UVB, 83% (10/12) of *Lats1*^−/−^ mice developed STSs, whereas no wild-type or heterozygous *Lats1^+^*^/−^ mice developed tumors (149).

#### RASSF1A

Epigenetic silencing of *RASSF1A* via hypermethylation of its promoter occurs in 20% (17/84) of adult STSs (189). (This study included six cases of RMS, which did not reveal *RASSF1A* hypermethylation.) *RASSF1A* silencing was especially common in leiomyosarcomas, and overall was associated with an increase in tumor-related death.

#### VGLL3

Like YAP, VGLL3 is a TEAD co-activator and has been identified as an inhibitor of terminal adipogenic differentiation, suggesting that it has a core role in mesenchymal cell fate (190). In a study of 404 adult STSs, recurrent amplifications of chromosomes 11q22 and 3p12, which contain genes for *YAP1* and Vestigial-like 3 (*VGLL3*), respectively, were identified in 10% of cases. Genomic amplification corresponded to overexpression of *YAP1* and *VGLL3* at the message level, and an increase in TEAD-associated genes. *In vitro* suppression of *YAP1* or *VGLL3* decreased cell proliferation and in the case of *VGLL3*, decreased migration (191). In a smaller study, analysis of eight NRSTS tumors identified 3p11-12 as a commonly amplified region of a ring chromosome 3 that was associated with high expression of *VGLL3* (192).

#### TAZ-CAMTA1 and YAP-TFE3 Fusions

Fusions between the *WWTR1* (gene name for TAZ protein) and *CAMTA1* genes were first noted in a NRSTS subtype termed epithelioid hemangioendothelioma (EHE) (193). EHEs are vascular sarcomas that can develop in bone, soft tissue, or visceral organs, and they demonstrate a clinical behavior intermediate between benign hemangiomas and high-grade angiosarcomas. Sequencing of two tumors identified the t(1;3) translocation between *WWTR1* and *CAMTA1*, and showed the fusion product to be under transcriptional control of the TAZ promoter. A larger study investigating 17 EHE tumors confirmed the translocation in all samples. The translocation and resulting transcript were not seen in epithelioid hemangioma and epithelioid angiosarcoma, morphologic mimics of EHE (194).

Subsequently, a *YAP1-TFE3* fusion product was identified in nine EHE samples that were morphologically different from the *WWTR1-CAMTA1* fusion-positive tumors (195). These findings were corroborated by two additional studies, the largest of which included 35 tumors and used a combination of IHC, FISH, and RT-PCR to validate *WWTR1-CAMTA1* fusion events in 33 cases and YAP1-TFE3 protein in two cases (196, 197). The oncogenic role of these signature fusions in EHE, or the role of Hippo signaling in vascular sarcomas, has not yet been established.

## Targeting Hippo Signaling for Therapy

Recognition of the importance of Hippo signaling in malignancy has led to preclinical studies aimed at targeting components of this pathway for anti-cancer therapy. Modeled genetic manipulation of Hippo components exhibit profound effects on tumorigenicity, which provides optimism that modulators of Hippo components could be effective in patients. Indeed, the Hippo cascade involves many protein–protein interactions that could serve as novel targets. For details on each potential modulator, see recent reviews in Ref. (201, 202).

### Small molecule modulators of the Hippo pathway

**Figure 3:**
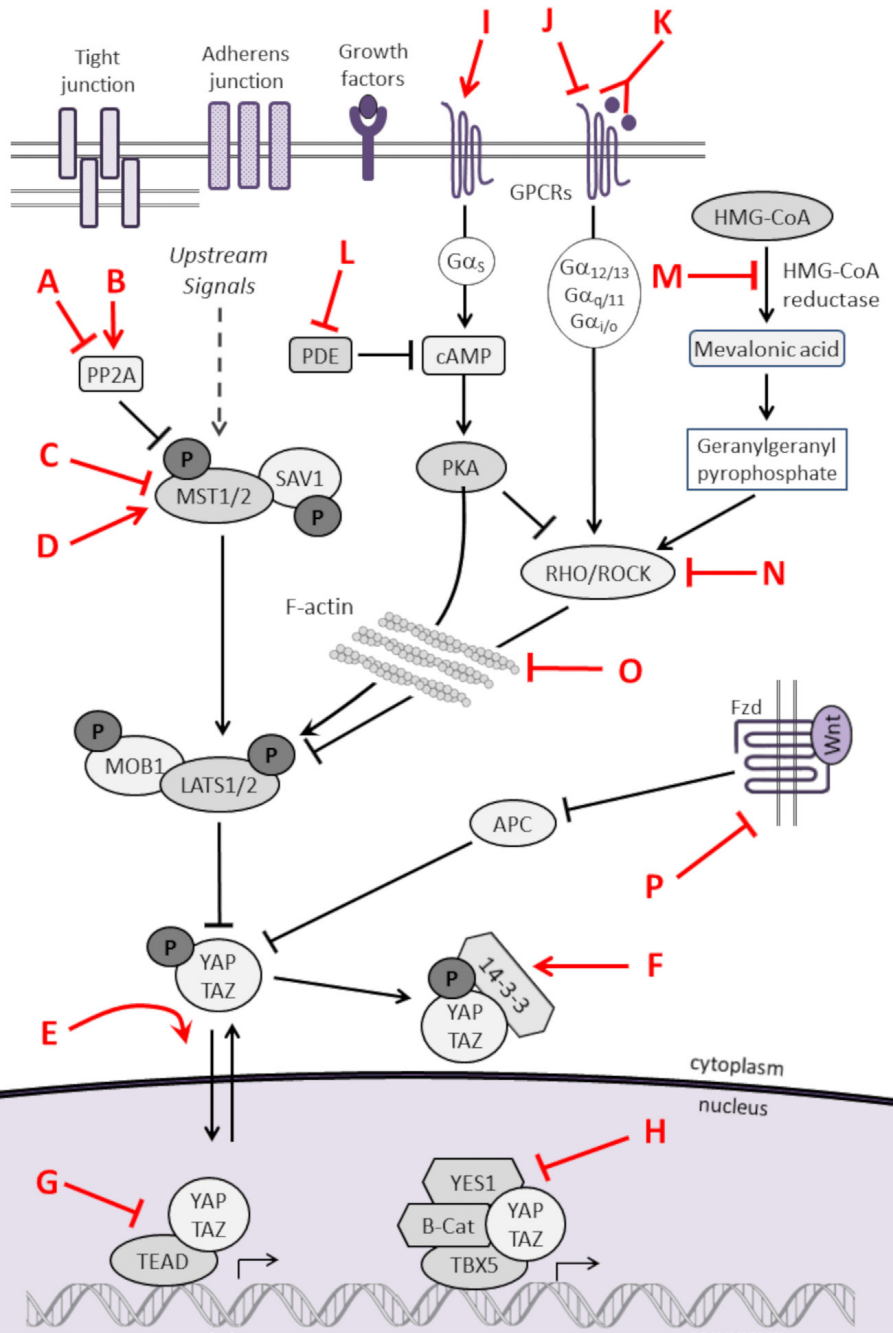
**Pharmacologic modulators of the Hippo pathway**. The Hippo cascade involves many protein–protein interactions that could serve as novel targets, and numerous pharmacologic compounds either directly or indirectly modulate Hippo activity. Some of the compounds activate Hippo components and others have an inhibitory role. While not all referenced studies have proven that modulation of upstream regulators result in concomitant changes in YAP or TAZ activity, these provide proof of principal that targeting Hippo signaling could be harnessed as a novel strategy to treat sarcomas. This is not an inclusive list, and other compounds are known to modulate Hippo components. Figure is modified with permission from Park et al. (202). Letters in Red correspond to the letters in the Key in Table 3.

As listed in Table 3 and highlighted in Figure 3, several pharmacologic compounds, that directly or indirectly modulate Hippo pathway activity, have been identified. However, a number of important challenges exist. First, while kinases are often excellent targets for small molecule inhibitors, the majority of kinases in the Hippo pathway are tumor suppressors, and restoring lost tumor suppressive function is not easily achieved. Moreover, and as highlighted here, aberrant hyperactivity of oncogenic YAP and TAZ is often seen in malignancy as a result of mutations in proteins from other signaling networks, even in the presence of intact upstream Hippo kinase activity. However, small molecules aimed at increasing YAP/TAZ phosphorylation-induced nuclear export and proteosomal degradation could be effective at reducing their activity.

**Table 3:** **Pharmacologic modulators of the Hippo pathway**.

Key	Compound	Mechanism	References
A	Fostriecin derivative	Inhibits PP2A	(210)
B	FTY720	Activates PP2A	(211)
C	9E1	Inhibits MST1 activity	(212)
D	C19	Activates MST/LATS	(213)
E	TM-25659	Modulates TAZ localization	(214)
F	Pyrrolidone 1	14-3-3 protein stabilizer	(215)
G	Verteporfin	Inhibits YAP-TEAD interaction	(49)
	Cyclic YAP-like peptide	Inhibits YAP-TEAD interaction	(205)
	VGLL4-like peptide	Inhibits YAP-TEAD interaction	(216)
	ABT-263, TW37	Inhibit BCL-xL (a YAP target)	(208, 217)
H	Dasatinib	Inhibits β-catenin-YAP-TBX5 complex	(58)
I	Epinephrine	Activates LATS through GPCRs	(29, 218)
	Dobutamine	Causes YAP phosphorylation	(219)
J	Phenoxodiol	SPHK1 inhibitor	(220, 221)
	BrP-LPA	LPA analog that blocks LPA receptors	(222)
	Thrombin	Acts on PARS to activate YAP	(223)
K	LT3015 Sphingomab	Monoclonal antibodies to LPA, S1P	(224–226)
L	Ibudilast	Inhibits PDE	(218, 227, 228)
M	Statins	HMG-CoA reductase inhibitors	(100, 101)
N	Y27632	RHO/ROCK inhibitors	(113, 116, 229)
	HA1077		
	Botulinum toxin C3		
O	Blebbistatin	F-actin destabilizers	(113, 115, 116)
	Cytochalasin D		(114–116)
	Latrunculin A/B		(113, 115, 116)
	ML7		(115)
P	WNT (or other pathway) modulators		(see Regulation Through Cross-Talk with Other Pathways and Hippo Modulation to Augment Other Pathway-Directed Therapies)

As such, inhibiting the activity of YAP/TAZ is the most obvious and presumably the most potent anti-cancer approach. Three porphyrin-related compounds were identified as top hits in a small molecule library screen of potential modulators for inhibiting the transcriptional activity of YAP *in vitro*. Verteporfin is a photosensitizer used clinically to treat patients with macular degeneration (203). Verteporfin binding to YAP alters YAP conformation to prevent it from binding to TEAD transcription factors. *In vivo* experiments in murine systems show verteporfin inhibits YAP-induced liver overgrowth by decreasing cell proliferation (49). *In vitro* treatment of retinoblastoma cells with verteporfin caused decreased cell proliferation and down-regulation of the pluripotency marker OCT4 (204). Other small molecule inhibitors, such as cyclic YAP-like peptides and TM-25659, have been developed to interfere with YAP/TAZ–TEAD interactions (205).

Another challenge is that the Hippo pathway is ubiquitously expressed and thus, systemic treatment may cause detrimental side effects. This is particularly important in the pediatric population, where normal growth and development in most tissues likely rely on intact Hippo signaling. Similarly, GPCRs, although relatively accessible to inhibition, have broad physiological functions. However, intestine-specific conditional *Yap1* knockout mice develop normally (206), implying that in some instances, YAP/TAZ may be dispensable for tissue development. YAP and TAZ are responsive to tissue-specific regulatory elements, presenting a theoretical possibility of targeting Hippo signaling in specific cells or tissues.

### Hippo modulation to augment other pathway-directed therapies

Evidence suggests Hippo-directed therapies may synergize with other targeted modulators. By serving as a parallel means of cancer cell survival, YAP promotes resistance to RAF and MEK inhibitors in *BRAF*/*RAS*-mutated tumors. YAP overexpression was observed in tumors harboring a *BRAF* mutation from patients with melanoma or NSCLC, and YAP expression levels inversely correlated to the patients’ initial response to RAF and MEK inhibition. Furthermore, YAP suppression enhanced MEK inhibition in murine xenografts of human NSCLC, melanoma, and pancreatic adenocarcinoma with *BRAF* or *KRAS* mutations (207, 208). Similarly, YAP upregulation of EGFR through a YAP–TEAD complex at the *EGFR* promoter has been shown to partly explain the reduced translational impact of EGFR inhibitors in cancer. Inhibition of the YAP–TEAD interaction using verteporfin results in decreased EGFR expression and enhanced chemosensitivity to 5-fluorouracil and EGFR inhibitors in mouse xenografts of esophageal cancer (209). Finally, mTOR inhibition with rapamycin results in decreased TAZ expression in hepatocellular carcinoma (90).

## Conclusion

The Hippo signaling pathway is an evolutionarily conserved tumor suppressor network important not only for proper cell, tissue and organ development, homeostasis, and repair, but it is also found dysregulated in many human cancers. While much of the early investigation on Hippo signaling in cancer was performed in epithelial malignancies, dysregulation of the Hippo pathway also occurs in sarcomas, cancers of mesenchymal origin. In a range of bone and soft-tissue sarcomas, Hippo signaling is commonly thwarted by upregulation of YAP or TAZ. However, genetic and epigenetic dysregulation of upstream core Hippo pathway members, and adaptor proteins has been noted. The role of Hippo signaling in mechanotransduction in both normal and cancerous mesenchymal cell behavior and fate provides additional insight into sarcoma biology. Further studies will be needed to clarify the underlying mechanisms of Hippo pathway dysregulation in specific sarcoma subtypes, providing a foundation upon which to develop successful therapeutic interventions.

## Conflict of Interest Statement

The authors declare that the research was conducted in the absence of any commercial or financial relationships that could be construed as a potential conflict of interest.

## Abbreviations

AMOT, angiomotin; APC, adenomatous polyposis coli; ARMS, alveolar rhabdomyosarcoma; β-TRCP, β-transducin repeat-containing E3 ubiquitin protein ligase; BMI-1, B-lymphoma Mo-MLV insertion region 1 homolog; BMP2, bone morphogenetic protein 2; BRAF, v-raf murine sarcoma viral oncogene homolog B; cAMP, adenylyl cyclase pathway; CD44, CD44 antigen; CDKN2A, cyclin-dependent kinase inhibitor 2A; ChIP-Seq, chromatin immunoprecipitation followed by high-throughput DNA sequencing; CRB, crumbs complex proteins; CTGF, connective tissue growth factor; dLats, large tumor suppressor (or warts); DMBA, 9,10-dimethyl-1,2-benzanthracene; DVL, disheveled; ECM, extracellular matrix; EGFR, epidermal growth factor receptor; EHE, epithelioid hemangioendothelioma; ERK, extracellular signal-regulated kinases; ERMS, embryonal rhabdomyosarcoma; EWS, Ewing sarcoma; FAT4, FAT tumor suppressor homolog 4; FOXM1, transcription factor forkhead box M1; GEMM, genetically engineered mouse model; GPCRs, G protein-coupled receptors; HMG-CoA, 3-hydroxy-3-methylglutaryl-coenzyme A; hTERT, telomerase reverse transcriptase; ID2, inhibitor of DNA binding 2; KIBRA, kidney and brain protein; LATS1/2, large tumor suppressor homolog 1/2; LLGL1, lethal giant larvae homolog 1; MAPK, mitogen-activated protein kinase; MARK1, MAP/microtubule affinity-regulating kinase 1; MCAT, muscle CAT elements; MDM2, mouse double minute 2; MEF2, myocyte enhancer factor 2; MEK, MAPK kinase; MMP-9, matrix metallopeptidase 9; MOB1, Mob kinase activator 1; MRFs, myogenic regulatory factor family; MSC, mesenchymal stem cell; MST1/2, serine/threonine kinases mammalian STE20-like protein kinase 1/2; mTOR, mechanistic target of rapamycin; MYCN, v-myc avian myelocytomatosis viral oncogene neuroblastoma derived homolog; Myf5, myogenic factor 5; MyHC, myosin heavy chain; MyoD, myogenic differentiation 1; NF2, neurofibromin 2 (or Merlin); NRSTS, non-rhabdomyosarcoma soft-tissue sarcoma; NSCLC, non-small cell lung cancer; OCT4, octamer-binding transcription factor-4; OS, osteosarcoma; p16INK4A, prototypic INK4 protein; PCNA, proliferating cell nuclear antigen; PDE, phosphodiesterase; PKA, protein kinase A; PKN1, protein kinase N1; PP1A, protein phosphatase 1, catalytic subunit, alpha isozyme; PP2A, protein phosphatase 2, regulatory subunit B, delta1; PPARγ, peroxisome proliferator-activated receptor gamma; RAF, v-raf murine sarcoma viral oncogene homolog; RAS, rat sarcoma viral oncogene homolog; RASSFs, Ras-association domain-containing family of proteins; RB1, retinoblastoma 1; RHO, rhodopsin; RMS, rhabdomyosarcoma; ROCK1, Rho-associated, coiled-coil containing protein kinase 1; RUNX2, runt-related transcription factor 2; SARAH domain, Salvador-Rassf-Hpo binding domain; SAV1, salvador homolog 1; SMADs, mothers against decapentaplegic proteins; Snai1/2, snail family zinc finger 1/2; SOX2, SRY (sex determining region Y)-box 2; STS, soft-tissue sarcoma; TAO-1, thousand and one amino acid protein 1; TAZ, transcriptional co-activator with PDZ-binding motif (or WWTR1); TCGA, The Cancer Genome Atlas; TEADs, TEA domain-containing sequence-specific transcription factors; TGFβ, transforming growth factor beta; TNF-α, tumor necrosis factor alpha; TP53, tumor protein p53; TP73, tumor protein p73; Twist1, twist family bHLH transcription factor 1; UVB, ultraviolet radiation B; VGLL3, vestigial-like 3; WNT, wingless-type MMTV integration site family; YAP, yes-associated protein 1.
